# Application of Polymers as a Tool in Crystallization—A Review

**DOI:** 10.3390/polym13162695

**Published:** 2021-08-12

**Authors:** Marcin Lemanowicz, Anna Mielańczyk, Tomasz Walica, Milena Kotek, Andrzej Gierczycki

**Affiliations:** 1 Department of Chemical Engineering and Process Design, Faculty of Chemistry, Silesian University of Technology, 44-100 Gliwice, Poland; tomasz.bartlomiej.walica@gmail.com (T.W.); kotekmilena8@gmail.com (M.K.); Andrzej.Gierczycki@polsl.pl (A.G.); 2 Department of Physical Chemistry and Technology of Polymers, Faculty of Chemistry, Silesian University of Technology, 44-100 Gliwice, Poland

**Keywords:** crystallization, pharmaceuticals, new materials, polymers

## Abstract

The application of polymers as a tool in the crystallization process is gaining more and more interest among the scientific community. According to Web of Science statistics the number of papers dealing with “Polymer induced crystallization” increased from 2 in 1990 to 436 in 2020, and for “Polymer controlled crystallization”—from 4 in 1990 to 344 in 2020. This is clear evidence that both topics are vivid, attractive and intensively investigated nowadays. Efficient control of crystallization and crystal properties still represents a bottleneck in the manufacturing of crystalline materials ranging from pigments, antiscalants, nanoporous materials and pharmaceuticals to semiconductor particles. However, a rapid development in precise and reliable measuring methods and techniques would enable one to better describe phenomena involved, to formulate theoretical models, and probably most importantly, to develop practical indications for how to appropriately lead many important processes in the industry. It is clearly visible at the first glance through a number of representative papers in the area, that many of them are preoccupied with the testing and production of pharmaceuticals, while the rest are addressed to new crystalline materials, renewable energy, water and wastewater technology and other branches of industry where the crystallization process takes place. In this work, authors gathered and briefly discuss over 100 papers, published in leading scientific periodicals, devoted to the influence of polymers on crystallizing solutions.

## 1. Introduction

The crystallization process has been known to humankind for thousands of years, since the vast majority of inorganic substances was and still is obtained in the form of crystals. The best example is the production of sodium chloride in ancient times by sea water evaporation using the Sun’s energy. A decade ago, more than 70% of solids manufactured by industry were obtained by crystallization or precipitation [1,2]. Despite this, the phenomenon of nucleation and crystal growth is not fully understood, resulting in some serious issues which need to be managed. For instance, the annual cost due to all types of fouling in industrialized countries is estimated at the level of 0.25% of their gross domestic product (GDP), which totals about EUR 35.75 billion for European Union in 2014 [3,4]. Thus, efforts are made to study the mechanism of crystalline matter deposition and as a result to reduce the cost of production and negative impacts on environment [5]. A much more serious challenge is related to the production of drugs, since human health and life may be directly threatened [6]. For example, in 1998 a new stable polymorph of ritonavir, a commercial product introduced to the market 18 months earlier, was detected which significantly reduced its solubility leading to a bioavailability issue [7,8]. The same scenario occurred with rotigotine whose sale was stopped in Europe and United States in 2008 [6]. These examples prove that the quality of crystalline products is of great importance both at the stage of manufacturing and storage.

At the present moment, two basic methods of crystallization are used in industry; namely, crystallization from solution and crystallization from melt. However, the later method will not be discussed here since it is out of scope of this paper. The basic parameters describing the crystallization process are supersaturation (supercooling), hydrodynamic conditions (mixing power), and crystal growth time. The condition of supercooling and supersaturation may be achieved by the cooling of the solution or by the evaporation of the solvent, respectively. Another aspect of crystallization is precipitation, in which the crystal nuclei are created due to a chemical reaction. In this case the hydrodynamic conditions play the dominant role in the control of the process.

The first stage of the crystallization process is named nucleation—a phenomenon leading to the creation of a stable cluster of molecules or ions, a nucleus which will later form the crystal [9]. Generally speaking, it may be divided into primary and secondary nucleation, whereas the primary nucleation may be considered as homogeneous and heterogeneous. The first case refers to the pure solutions in which no crystals are present. The population of clusters is created—some of them are short-lived entities which will eventually break, but some of these clusters will create the critical nucleus leading to the formation of crystals. Simultaneously it is worth emphasizing that homogeneous nucleation is rare and hard to accomplish. On the other hand, the second case refers to the solutions in which impurities are present. Importantly, the foreign substance which inhibits the nucleation in one system may induce this process in another one. This is a principle that will be broadly used in the research presented in this paper. The term secondary nucleation is used for description of the nucleation which takes place in a solution in which the crystals of solute are already present. Such a phenomenon occurs more readily compared to the primary, homogeneous nucleation. Therefore, often in industrial practice the seeding process is used during which the crystals are added deliberately to the system. In the case of crystallization realized in agitated vessels, the secondary nucleation may occur due to the attrition and breakage of already formed crystals due to the hydrodynamic conditions [9].

The second stage of the crystallization process is crystal growth. One may find multiple families of theories governing this phenomenon. For example: surface energy theories, adsorption layer theories, kinematic theories, and diffusion-reaction theories [9]. Although this topic will not be discussed here an interested reader may find multiple works dedicated to this phenomenon, such as a review of Chernov [10] or the more recent work of Lovette et al. [11]. However, from the crystal growth another important issue emerges, namely polymorphism. Polymorphism is common in crystallization. Since the polymorphs have a different type of lattice or spacing of lattice points, they may exhibit significantly differing properties (density, solubility, reactivity etc.) and therefore change, for example, their bioavailability. It is also worth emphasizing that one should not confuse polymorphism with different crystal habits [9].

A large group of papers are focused on efforts to elucidate and describe the mechanisms governing complex processes of crystallization in the presence of polymers. For example, the selective production of crystalline polymorphs, an outstanding issue in solid-state chemistry, is of great importance in the industry. Generally speaking, the polymers may be used for confined crystallization in which the nucleation occurs within the polymeric mesh of gel. They can be also used for the creation of functionalized surfaces or specific substrates which will promote the nucleation process. On the other hand, free macromolecules present in the solution may shield specific surfaces of crystals or even inhibit the nucleation process, thus influencing the subsequent crystal growth. Finally, one can also find many publications in which polymers are used in ways which fall outside the above division.

## 2. Basic Research

### 2.1. Confined Crystallization

Ma et al. documented a specific method for combining calcium ions and alginate molecules in mineralization [12]. The presented results could contribute to the understanding of the mineralization process of calcium carbonate (CaCO_3_) in hydrogel systems. The same research group reported experiments dealing with CaCO_3_ crystals’ growth in silk fibroin hydrogel by a simple ion diffusion method [13]. The study gave a better explanation of the influence of silk fibroin concentration and its structure on CaCO_3_ crystals’ growth. Nindiyasari et al. investigated the influence of the porosity of the growth medium on the crystallization of CaCO_3_ in hydrogels with different gelatin solid contents [14], whereas Kosanović et al. crystallized CaCO_3_ polymorphs in alginate and xanthan hydrogels in which the degree of entanglement was altered by the polysaccharide concentration [15]. Crystallization experiments performed in alginate hydrogels indicated the initial formation of a mixture of calcite, vaterite and amorphous CaCO_3_. In turn, Yokoi et al. tested the formation of single or two coexisting crystalline phases of calcium phosphate (Ca_3_(PO_4_)_2_), hydroxyapatite, hydroxyapatite/dicalcium phosphate dehydrate or octacalcium phosphatein polyacrylamide hydrogels containing phosphate ions by diffusion of calcium ions from calcium nitrate solutions [16]. They found that the dense hydroxyapatite layer and hydroxyapatite/dicalcium phosphate dehydrate layer prevented the diffusion of calcium ions from the calcium nitrate solution, thus inhibiting the formation of Ca_3_(PO_4_)_2_ in the gel phase. Velásquez-González et al. discussed the theory and practice used for the preparation of polymorphs, use of gels into crystallogenesis of different substances, particularities of protein crystal polymorphism and also modern trends in gel growth for high-resolution X-ray crystallography [17]. In turn, Choi et al. provided evidence for synthetic biomimicry similar to bio-polymerization mechanisms to steer disorder-to-order transitions via solvent plasticization-like behavior [18]. In addition, Diao et al. demonstrated a new method to control nucleation with polymer microgels [19]. They found that the polymer microstructure had a significant impact on nucleation kinetics. Moreover, they demonstrated that by tuning the polymer-solute interactions, solute nucleation kinetics was significantly promoted [20]. Finally, Li et al. studied crystallization of two types of zeolites in glutaraldehyde crosslinked chitosan hydrogels [21]. They showed that chitosan hydrogen is a promising space-confinement medium for the synthesis of zeolite nanocrystals.

### 2.2. Crystallization on Polymeric Surfaces/Nucleation Induction

Now, the application of functionalized surfaces as well as synthesized substrates for nucleation induction will be discussed. For example, Curcio et al. investigated the influence of the morphological parameters of microporous poly(vinylidene fluoride) membranes on the heterogeneous nucleation rate of hen egg white lysozyme crystals [22]. They obtained a mathematical correlation between the energy nucleation barrier, membrane porosity and the contact angle between protein solution and polymeric substrate. On the other hand, Lin et al. studied the surface crystallization of calcium sulfate dihydrate (gypsum) on a series of polymeric surfaces using a quartz microbalance system [23]. They discovered that the extent of surface mineral scaling was the combined result of the rate of nucleation and crystal growth kinetics. In turn, Patel et al. in their paper described a new method called Predicting Efficacy Through Intermolecular Interactions (PETI) for predicting the effectiveness of different polymer surfaces in promoting heterogeneous nucleation [24]. Solomos et al. crystallized diphenylurea via slow evaporation from a range of solvents [25]. The experiments demonstrated the utility of template-directed sublimation methods as a way to obtain metastable phases with high phase purity. Hutfles et al. tested the influence of various commercial polymer films on the crystallization of CaCO_3_ from a supersaturated mixture containing other salts [26]. Such methodologies and data can be useful for designs of crystallizers and mixing devices. Finally, it is worth mentioning the crystallization of polymers with the use of other polymers. At this point it has to be emphasized that this research topic is extremely popular and one may easily find multiple papers dedicated to this phenomenon. Therefore, here the work of Xing et al. is presented just as an example of such research. These authors in their experiments proved that the crystallization rate of poly(ethylene terephthalate) was significantly accelerated and melt-crystallization temperature was increased with the addition of ionomers of poly(styrene-alt-maleic anhydride)/organic montmorillonite nanocomposites [27]. The nanocomposites acted as heterogeneous nucleating agents which enhanced the crystallization process. According to these authors the synergy was achieved for a combination of ionomers and organic montmorillonite platelets.

### 2.3. Crystal Surface Screening/Nucleation Inhibition

Next, the impact of free macromolecules in solution onto the crystallization process will be described. Yue et al. in their experiments achieved dumbbell-like ZnO hollow architectures by a simple solution-based method using poly(sodium 4-styrenesulfonate) as a crystal growth modifier [28]. In this case, the sheet-like particles were formed due to the presence of polymer. The subsequent formation of crystals was the result of dissolution and the Ostwald ripening process. The ZnO architecture showed unique photoluminescence characteristics at room temperature, suggesting potential usage in optoelectronic applications. Miculescu et al. presented the generation of zinc oxide nanocrystalline structures in the presence of two water-soluble polymers with totally different chemical structures [29]. The method allowed the obtaining of hierarchical ZnO nanostructures in a controllable manner. The novelty of the paper is in the electrochemical synthesis of inorganic particles in the presence of the water-soluble polymers. In turn, Zhang et al. prepared molecularly imprinted polymers in precipitation polymerization, using oleanolic acid as template, acrylamide as functional monomer, ethylene glycol dimethacrylate as cross-linker and azobisisobutyronitrile as initiator in a mixture solvent of chloroform and methanol [30]. The obtained results indicated that molecularly imprinted polymers showed good specific adsorption properties and the ability to induce crystallization of oleanolic acid in supercritical CO_2_. Preda et al. synthesized lead sulfide micro- and nanoparticles by a precipitation reaction of lead nitrate with thioacetamide in water solutions of polyacrylamide, polyvinyl alcohol, polyethylene glycol and poly(*N*-vinyl pyrrolidone) (PVP), respectively [31]. They obtained polydisperse lead sulfide particles with a cubic shape in case of polyacrylamide and polyethylene glycol, while monodisperse cubic lead sulfide crystallites in the presence of poly(vinyl alcohol) (PVA) and PVP. Pelin et al. investigated the maleic acid copolymers role on calcium orthophosphates’ crystallization at low temperatures [32], whereas Lu et al. investigated the non-isothermal crystallization behavior of copper/poly(ethylene oxide)/low density polyethylene composite by using differential scanning calorimetry [33]. The results showed that the crystallization of low density polyethylene was retarded by the addition of poly(ethylene oxide) with a higher molecular weight and accelerated by cooling with a larger cooling rate. Arioglu-Tuncil et al. used solid dispersions of thiamine chloride hydrochloride in the presence of variety of polymers with different physicochemical properties in order to investigate the efficacy of polymers as crystallization inhibitors [34].

### 2.4. Other Aspects of Basic Research

Finally, the examples of research which fall outside abovementioned categories will be presented. For example, Sinek et al. considered the employment of stimuli-responsive polymer phase transition for triggering the nucleation process within a solution [35]. They hypothesized that the sudden appearance of hydrophobic particles could destabilize the metastable solution. Such an approach could eliminate the stochastic nature of the crystallization process. In their research, a poly(acrylic acid)—KCl water solution was used. So far, they have proved that it is possible to initiate the polymer’s phase transition near the solubility curve of dissolved salt. Beyer et al. investigated the crystallization of highly cross-linked polystyrene particles dispersed in 2-ethylnaphtalene and their mixtures with non-adsorbing low molecular weight polystyrene [36]. They observed that the increased polymer concentration shifts the balance between crystallization pathways giving the possibility to tune the amount of wall-based crystals. Marinović-Cincović et al. performed a comparative kinetic analysis of non-isothermal crystallization for europium ions (Eu^3+^) doped Zn_2_SiO_4_ samples, prepared by the polymer-induced sol–gel method [37]. They found that the probability for two-dimensional crystal growth increases, especially at higher heating rates, and also that the substitution of zinc ions by europium ions in Zn_2_SiO_4_ matrix can lead to some crystal defects. Sun et al. applied poly(vinyl butyral) as a nucleating agent into the monomers prior to polymerization of poly(butylene succinate) for accelerating crystallization and enhancing mechanical properties [38]. The nucleated poly(butylene succinate) exhibited a considerable improvement in mechanical properties. Liu et al. investigated the isothermal crystallization behavior of water at −30 °C in poly(vinyl methyl ether) aqueous solution with a poly(vinyl methyl ether) concentration in the range of 40–60 wt% [39]. The results showed that the crystallization rate decreases with increasing poly(vinyl methyl ether) concentration. It is interesting that the so-called “unfrozen bound water” can be frozen slowly when poly(vinyl methyl ether) aqueous solution is annealed at a suitably low temperature. Mandal et al. presented a multi-scale simulation method for modeling crystal growth in the presence of polymer excipients [40]. The proposed model can be used to study the effect of additives, such as polymers, on the inhibition of crystal growth by polymers. Yan et al. applied the temperature-dependent aggregation features of selected chemical compound to separate nucleation and growth processes [41]. Olmstead and Ghiassi in their review paper presented a general survey of up-to-date knowledge of various aspects of crystal growth [42]. Bakshi et al. [43] carried out aqueous-phase synthesis of lead sulfide nanocrystals and microcrystals using cationic twin-tail surfactants. They observed that the increase in hydrophobicity, by introducing another tail in the basic structure of dodecyltrimethylammonium bromide, significantly controlled the shape and size, and lead to the formation of, well-defined nanocubes and spheres.

### 2.5. Discussion and Conclusions

**Figure 1:**
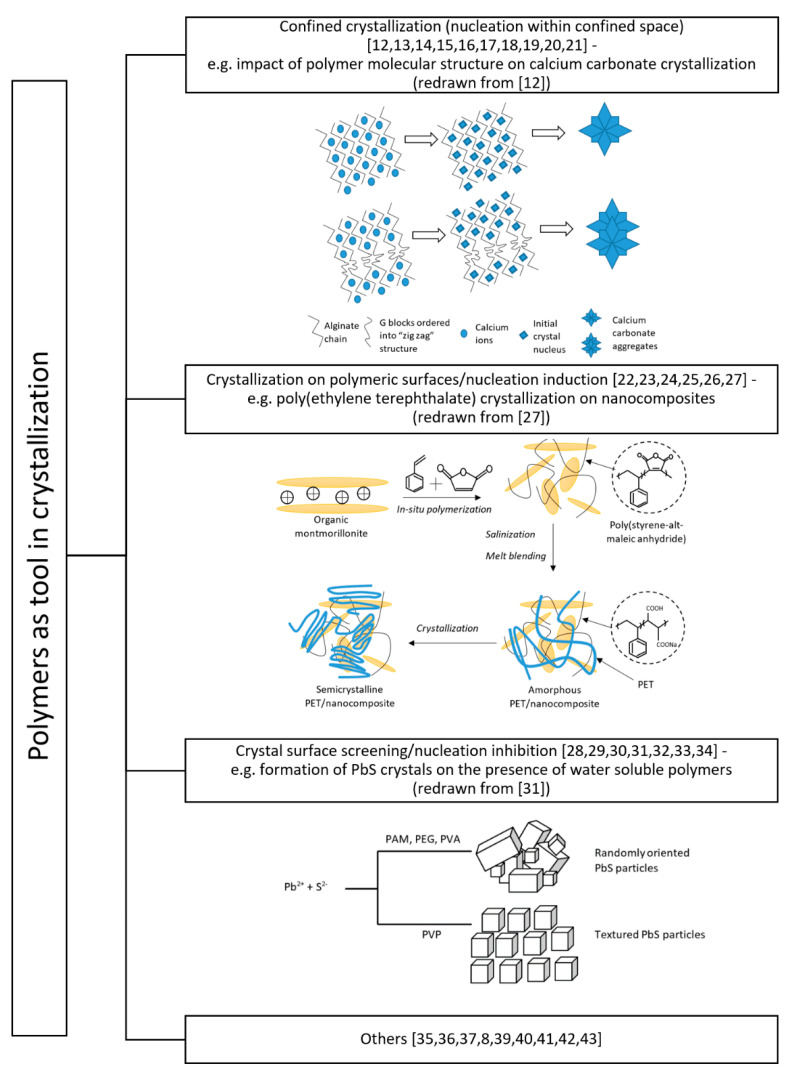
The classification of polymers’ usage as a tool in the crystallization process.

The first paragraph of this paper indicates that polymers offer a promising approach for the rational design of materials for controlling nucleation from solution (Figure 1). The confined crystallization with the application of polymeric matrixes becomes more and more popular since it allows one, for example, to mimic biological systems. The influence of gels on crystal growth depends on the type of gel being used. A decrease in solubility of any solute in the liquid may occur if the solvent interacts extensively with the polymers. Hence, the nucleation in gels in these cases occurs at relatively low supersaturations. The pores of gels are often referred as microreactors. Due to the specific conditions within such “vessels” a control over polymorphism may be achieved. This fact opens totally new routes of drug synthesis. However, the results obtained with the use of polymer microgels indicated that systematic studies are necessary in order to quantitatively clarify factors influencing, for example, surface mineral scaling crystallization.

Other experimental results on polymer-solute interactions provided insights into nucleation at interfaces and enabled a rational designing of the nucleation of molecular crystals from solution (Figure 1). The lack of understanding the influence of surface properties on nucleation is an important problem in the design of crystallization processes. Unfortunately, current approaches are often empirical and lack any predictive capability. Usually, each system has to be treated as a separate case. However, a highly novel approach, PETI (Predicting Efficacy Through Intermolecular Interactions), presented in some papers, could be a tool in the rational design of polymer surfaces and the control of heterogeneous nucleation. This approach utilizes the Cambridge Structural Database (CSD) to determine the possibility of forming an intermolecular interaction between solute chemical moieties and polymer surfaces [44]. Moreover, one may also find other studies dedicated to the simulation and mathematical modeling of the crystallization mentioned above, e.g., a coarse-grained model which was successfully used for analysis of melt crystallization or crystallization with additives.

Simultaneously, knowledge of the relationship between crystallization and phase separation is important to optimize solution processing performance of polymer blends. Crystal nucleation and crystal growth are coupled and it is sometimes difficult to distinguish their effects on phase separation. Therefore, many experimental works are devoted to these issues. Concluding, it has often been repeated that crystal growth is an art. Direct control of nucleation and crystal growth in a crystallization process is difficult to achieve but the use of assisting polymer additives offers many potential benefits to the food, chemical, and pharmaceutical industries (Figure 1).

## 3. Present and Future Perspectives of Applications of Crystallization Phenomena in the Presence of Polymers

Although crystallization, for decades, was considered an empirical science, now, based on the advantages of research on nucleation and growth theory at the more precise molecular level, it turns to theory-based science and practice. The pharmaceutical industry is one of the examples where crystallization technology is the most important issue since it governs solid-state phase transformation and stability of pharmaceuticals. Therefore, vast publications related to this field concern groundbreaking discoveries about the effect of the polymer presence on co-crystals, polymorphs, and solvates of drugs. In addition, there are also examples of publications in the context of the phenomenon of crystallization in the presence of polymers, where researchers focused on the testing and production of new materials used in various branches of medicine and technology, such as renewable energy, detection, optoelectronics and applications in wastewater treatment.

### 3.1. Polymers Used in Pharmaceuticals to Prevent the Nucleation of Crystalline Drugs or Precipitation of Amorphous Drugs

#### 3.1.1. Antiinflammatory Drugs

There is a group of papers concerning the improvement of the production technology of different model drugs using polymer additives. The first direction for utilization of polymers is preventing the nucleation of crystalline drugs or precipitation of amorphous drugs. Velasco et al. tested homopolymers and copolymers of *N*-ethylmorpholine methacrylamide (EMA) and *N*,*N*-dimethylacrylamide (DMA) hydrogels as matrices for aqueous ibuprofen (nonsteroidal anti-inflammatory drug) release to prevent possible damage of the mucous membrane of the stomach [45]. The most promising results were obtained for EMA hydrogels which were able to prevent crystallization of ibuprofen at all pH values. Crystallization of ibuprofen with the addition of poly(acrylic acid) (PAA), PVP or poly(4-vinylphenol) (PVPh) as polymeric additives was also studied by S. Lee et al., showing that PVP was the most effective additive that prevented the crystal nucleation and allowed the sustained release of ibuprofen [46]. In another paper, the effect of functionalized poly(chloromethylstyrene-*co*-styrene) on the stability in amorphous solid dispersions of drugs was studied [47]. The authors found that hydrogen bonding between functionalized polymers and nabumetone (non-steroidal anti-inflammatory drug) can improve stability against crystallization. They also found that supersaturation maintenance for ethenzamide (analgesic and anti-inflammatory drug) was improved by increasing the hydrophobicity of dissolved poly(*N*-hydroxyethyl acrylamide) in an aqueous solution [48].

#### 3.1.2. Other Drugs

Alonzo et al. studied the ability of hydroxypropylmethyl cellulose (HPMC) to act as an inhibitor of the nucleation and crystal growth of felodipine (a drug used to treat high blood pressure) [49]. It was found that the presence of ppm levels of pre-dissolved HPMC could inhibit both the nucleation and growth of felodipine crystals. Moreover, the authors proved that delaying nucleation was much more crucial to the stabilization of supersaturated solution than hindering crystal growth. Guan et al. investigated the influence of the drug-alginate miscibility on maintaining drug supersaturation using three different model drugs: lovastatin (statin used to prevent and treat coronary heart disease), indomethacin (a drug used to reduce fever, pain, stiffness, and swelling from inflammation) and itraconazole (a drug for fungal infections), and they concluded that alginate could be used as a potential crystal growth inhibitor [50]. Hong et al. studied the precipitation inhibitory effect of HPMC and methylcellulose polymers using griseofulvin (applied in fungal infections) as a poorly water-soluble model drug [51]. Ozaki et al. studied the impact of water-soluble polymers on the supersaturation behavior of amorphous pharmaceuticals [52]. HPMC, PVP and Eudragit L-100 were used as polymers and griseofulvin or danazol (a drug used in the treatment of endometriosis and some benign breast disorders) as model drugs. The results of experiments demonstrated that the polymers contributed to drug supersaturation by inhibiting both nucleation and growth but the effect of the polymers was drug dependent.

Although most studies concern the influence of a predetermined polymer on the selected drug, there are also some articles which describe a more complex approach to the issue. Fornells et al. tested the ability of different polymers of vinylpyrrolidone and a copolymer of vinylpyrrolidone and vinylacetate for their impact on the supersaturation of sixteen different drugs [53] using the CheqSol method. They observed that, contrary to basic drugs, acidic compounds displayed enhanced solubility in the presence of PVP. Chavan et al. provided an overview of different mechanisms by which cellulose derivatives inhibit the crystallization of drugs in the solid state and from a supersaturated solution [54].

### 3.2. Polymers Used in Pharmaceuticals to Control the Nucleation of Crystalline Drugs or Precipitation of Amorphous Drugs

#### 3.2.1. Antiinflammatory Drugs

Polymers can be applied in both directions—preventing the nucleation/precipitation as well as for direct control of the crystallization/precipitation processes. H. Lee and J. Lee showed that PVP-induced crystallization of celecoxib (nonsteroidal anti-inflammatory drug) improved its bioavailability, stability and processability [55]. Celecoxib as a model drug was also used in experiments presented by Sodhi et al. Using the microarray plate method they tested the inhibition performance of HPC, HPMC and PVP on the crystallization of celecoxib [56]. The applicability of the microarray plate method for quantitative estimation of precipitation kinetics for poorly soluble drugs was demonstrated. Sharma and Trout reported the results of a simulation showing that paracetamol aggregation is predominantly governed by the size of the pores of poly(ethylene glycol diacrylate) and the polymer-paracetamol interactions play a secondary role [57]. Pfund et al. described the experiments in which the tailor-made copolymers of *N*-hydroxyphenyl methacrylamide and styrene decreased the induction time for paracetamol crystals appearance [58]. In their paper Sudha et al. proposed a new method of inducing the preferred mono- or ortho-polymorphic forms of paracetamol through the swift cooling of boiled aqueous solution in the presence of selective polymers such as nylon 6/6 (C_12_H_22_N_2_O_2_)_n_, polypropylene (C_3_H_6_)_n_ and polyvinylchloride (C_2_H_3_Cl)_n_ [59]. Song et al. investigated the polymer-induced heteronucleation method to test the effect of nylon 66, PET and polypropylene films on the crystal growth of paracetamol and found different induction times for different polymers [60]. Stojakovic et al. utilized biocompatible 2-hydroxyethyl cellulose thin films to enhance the nucleation rates of paracetamol and found that flat polymer films without nano-imprinting enabled the crystallization of paracetamol two times faster than bulk crystallization [61]. Frank and Matzger reported a methodology for comparing nucleation efficiencies of different functionalities on polymer heteronuclei of uniform topology (functionalized cross-linked poly(chloromethylstyrene)) that best accelerate nucleation of paracetamol [62]. The presented study shows that the increased hydrophobicity of the resin functionalized with methylated paracetamol results in faster heteronucleation of the drug, as evidenced by its higher average induction time. Price et al. reported the methodology to control crystal polymorphism through the use of diverse polymer heteronuclei including both commercially available and synthesized cross-linked polymers [63]. The proposed technique was successfully demonstrated in pharmaceuticals such as paracetamol, sulfamethoxazole (an antibiotic used against bacterial infections) and carbamazepine (an anticonvulsant drug). This new approach offers the advantage of producing multiple polymorphs.

Tan et al. demonstrated a new approach for designing and fabricating biocompatible PVA films that can enhance nucleation rates and enabled polymorph selection of small-molecule compounds such as aspirin (medication used to reduce pain, fever, and inflammation) and indomethacin [64]. The research shows that PVA accelerated the heterogeneous nucleation rates of drugs. Cheng et al. tested three different polymers, i.e., PVP, HPMC, and Kollidone, to assess their impact on the nucleation and crystal growth of indomethacin from supersaturation solutions [65]. Diao et al. patterned polymer films based on PAA crosslinked with DVB with nanopores of various shapes and found that spherical nanopores 15–120 nm in diameter hindered the nucleation of aspirin crystals, whereas angular nanopores of the same size promoted it [66]. Their results can be used in the control of pharmaceutical polymorphism and for the regulation of the crystallization of fine chemicals. They used aspirin and indomethacin as model compounds. Diao et al. found that the presence of nanoscopic pores on certain polymer surfaces, i.e., poly(4-acryloylmorpholine) (PACM) and poly(2-carboxyethyl acrylate) (PCEA), each cross-linked with divinylbenzene, led to order-of-magnitude faster aspirin nucleation rates when compared with surfaces without pores [67].

Poornachary et al. investigated the effects of polymeric additives HPMC and PVP used in pharmacy to stabilize crystalline suspensions on the growth kinetics of naproxen (non-steroidal anti-inflammatory drug [68]. The influence of PVP on the crystal growth of the drug was explained based on hydrophobic—hydrophilic intermolecular interactions occurring at the crystal—solution interface. Two years later, they released a publication on the utilization of the molecular dynamics simulations to model the interactions among PVP, naproxen and ethanol/water mixture occurring at the crystal/solution interface [69]. The presented results were consistent with experimental observations and enabled the understanding of the mechanism behind the anisotropic growth behavior of a naproxen crystal in the presence of HPMC and PVP additives.

#### 3.2.2. Drugs Affecting Blood Pressure 

Pataki et al. performed carvedilol (medication used to treat high blood pressure) precipitation by combined antisolvent-cooling crystallization in the presence of PVP with different molecular weights [70]. A small amount of PVP adsorbed on the crystal surface affected positively the crystal form and induced excellent flowability of the products. Xia et al. investigated the amorphous-to-crystalline transformation of nitrendipine (drug used in the treatment of blood hypertension) in a medium containing PVA and PEG [71].

Eral et al. proposed a method for producing crystals of poorly water-soluble active pharmaceutical ingredients embedded in a polymer matrix, i.e., crosslinked alginate [72]. They presented a material-based approach to enable control over the crystal size and morphology of a model hydrophobic drug—fenofibrate (drug decreasing the level of cholesterol). The used method avoided excessive energy input and high saturation levels that could lead to amorphous forms or the appearance of metastable crystal phases. Moreover, it enabled control over drug loading. Authors claimed that due to the biofriendly nature of alginate hydrogels, adjustable submicrometer crystal size and high loading capacity of the drug, the proposed composite material could potentially serve as a final drug formulation which could be produced in a continuous manufacturing process. In another paper Eral et al. investigated the usage of biocompatible alginate hydrogels as a material for crystallizing and encapsulating paracetamol and fenofibrate as model active pharmaceutical ingredients [73]. They found that the incorporation of emulsion droplets inside hydrogels enables the high loading of the hydrophobic pharmaceutical ingredients leveraging the high solubility of hydrophobic drug in the dispersed emulsion droplets. Badruddoza et al. described a new bottom-up approach for producing and formulating nanocrystals of fenofibrate using core—shell composite hydrogel beads composed of an alginate core with a PVA shell [74]. The method enables the controlled crystallization and formulation of hydrophobic crystalline nanomaterials that can replace energy intensive top-down processes in traditional manufacturing.

Bae and J. Lee investigated the influence of polyethylenimine (PEI) on the particle morphology of crystals during the crystallization of eprosartan (a drug used in high blood pressure treatment) [75]. The presence of PEI during the crystallization induced the self-assembly of small primary crystals into larger aggregates without changing the crystal polymorph, which improved the properties of drug crystals.

#### 3.2.3. Antibiotics

Munk et al. studied the isotactic and atactic poly(*N*-isopropyl acrylamide) (PNIPAM) as an additive in evaporative crystallization of nitrofurantoin (drug in the urinary tract infections treatment) [76]. They found that slower nucleation and growth rates of the crystals were observed regardless of the molecular weight or stereoconfiguration of the PNIPAM. Czyzewski et al. in their experiments used a polymer additive such as HPMC or PVP to successfully reject impurity of sulfonamide based drug which cannot be removed via conventional crystallization [77]. Frank et al. using pyrazinamide (an antibiotic used to treat tuberculosis) and hydrochlorothiazide (diuretic used to treat blood hypertension and edema) as model pharmaceutical, demonstrated that the rate of crystallization can be controlled by polymer solubility present in the solution [78]. The authors presented results for insoluble polymers which accelerated crystallization—functionalized Merrifield resin (MFR), and a cross-linked poly(styrene-*co*-chloromethylstyrene) (P(S-*co*-ClMS))—as well as for soluble polymers to inhibit crystallization-functionalized poly(hydroxyethyl acrylamide-*co*-chloromethylstyrene) (PHEAM). In addition to the previously mentioned work from Price et al., the crystallization of sulfamethaxazole was also investigated by Palomero et al., who observed that organogels prepared from carboxylated nanocellulose (CNC) in the presence of cationic surfactant can be used as novel crystallization media for pharmaceutical solid form control [75]. Torres-Moya et al. showed that organogel based on f 2*H*-benzo[*d*]1,2,3-triazole derivative can be used in the crystallization of sulfathiazole, inducing a change in the polymorphic form of the drug [79]. 

#### 3.2.4. Diabetes

Shi et al. investigated the impact of amorphous PVP and semicrystalline (polyethylene glycol 6000, polyethylene–polypropylene glycol 188) polymeric additives on the crystallization behavior of pioglitazone solid dispersions (drug applied in the treatment of diabetes mellitus) [80]. All polymers used in experiments were observed to reduce crystal growth rates of pioglitazone compared with the drug alone. However, amorphous polymers were more effective at inhibiting crystallization rates, while semicrystalline polymers were less effective. Gao and Olsen proved in their experiments that the diblock copolymer, poly(ethylene glycol)-*block*-poly(lactic acid) (PEG-*b*-PLA) modulated the crystal growth of tolazamide (drug applied in the treatment of diabetes mellitus) [81]. They found that polymers capable of forming strong hydrophobic and van der Waals interactions might be more effective in inhibiting crystallization of poorly water-soluble and hydrophobic drugs in aqueous media than those with hydrogen-bonding capacities. 

#### 3.2.5. Other Drugs

Choi et al. reported studies on biomimetic polymer-directed crystallization techniques applied to the crystallization of atorvastatin (a popular drug belonging to statins) [82]. In the presence of poly(ethylene glycol) (PEG), polyethylene imine (PEI), hydroxypropyl cellulose (HPC), polyethylene oxide-polypropylene oxide triblock copolymer (PEO–*b*-PPO–*b*-PEO) and PAA, nucleation and growth of atorvastatin at improved conditions led to obtaining composite crystals with significant polymer contents and unusual characteristics, such as a decreased melting point, improved stability, and sustained-release patterns. 

Rahim et al. presented a natural polyphenol-based supramolecular metallogel system for active pharmaceutical ingredients crystallization such as caffeine, carbamazepine and piroxicam (an anti-inflammatory drug) [83]. Advantages of this system include ease of preparation, use of inexpensive components and the ability to incorporate diverse additives. Diao et al. reported the use of polymer microgels, formed by cross-linking polyethylene glycol diacrylate of varying PEG subchain molecular weights, for achieving selective crystallization of carbamazepine polymorphs [84]. They showed that polymer microgels with tunable microstructure are promising materials for controlling crystal polymorphism.

Chen et al. presented a novel method for continuous polymer coating of griseofulvin crystals based on solid hollow fiber cooling crystallization [85]. The Eudragit RL100 polymer precipitated from the solution and coated the suspended crystals due to rapid temperature reduction and heterogeneous nucleation. 

Shi et al. studied in detail the effect of ethyl cellulose on the recrystallization of agomelatine (a drug used in patients suffering from a depressive disorder associated with insomnia) from a molten state [86]. 

Finally, it is worth mentioning several review papers which, in depth, tackle the subject of novel solutions regarding crystallization of drugs in the presence of polymers. For example, Parambil et al. wrote an overview on the use of template-induced crystal nucleation for formulating drug substances and enabling nucleation and polymorphic control during continuous manufacturing of active pharmaceutical ingredients [87]. Thakore et al. in their review paper presented recent findings in the primary heterogeneous crystallization with specific emphasis on its pharmaceutical applications [88]. Sood et al. discussed how excipients or formulation variables such as type of solvent, presence of hydrophilic adjuvants, surfactants, dyes and colorants improve stability, modify release and improve physicochemical properties of dosage forms [89].

#### 3.2.6. Discussion and Conclusions

Crystallization is one of the most important unit operations in the quality control of pharmaceutical products. Specific molecular (i.e., polymorphism) and particulate (i.e., particle size and crystal habit) properties of pharmaceuticals can be obtained by their controlled crystallization. Moreover, these molecular and particulate properties govern the manufacturability, stability and biopharmaceutical performance of the drugs. Current methods for controlling critical crystal size and morphology are inefficient and often lead to undesirable solid states such as metastable polymorphs or amorphous forms. To avoid this, an approach for producing crystals of a poorly water-soluble pharmaceutical compound embedded in a polymer matrix is proposed. Polymer-induced crystallization creates opportunities for convenient pharmaceuticals engineering, allowing consistent improvements in bioavailability and processability. Furthermore, the technique of polymer-directed crystallization seems to be promising for the improving properties of drug crystals. The researchers indicated different ways to rationally design the geometry of heterogeneous surfaces to enhance the nucleation of small-molecule organics. The crystallization of active pharmaceutical ingredients can be carried out using supramolecular gel media. As a result, control over the crystal properties like size, morphology, and polymorphism was gained. Moreover, it has been shown that the utilization of polymers offers additional advantages such as drug solubilization, crystallization inhibition and improvement in the release patterns of drugs.

### 3.3. New Materials

#### 3.3.1. Water Treatment Agents

Polymer-based water treatment agents have recently received much more attention due to their environmental friendliness, widespread availability, and favorable structural features. Wang et al. investigated a novel low-phosphorus copolymer that strongly inhibited calcium sulfate dihydrate scale formation in industrial cooling water treatment [90]. Zhang et al. compared the efficiency of three polymeric antiscalants: poly(aspartic acid-citric acid) (PAACA) copolymer, poly(maleic acid) (PMA), and a compound inhibitor being the mixture of two earlier mentioned ones [91]. The compound inhibitor showed a better inhibition performance than the above two kinds of monomers for calcium phosphate scale prevention. Zhao et al. tested poly(citric acid) (PCA) polymer for CaSO_4_ scale prevention [92]. They found that the polymer hinders the CaSO_4_ scale crystal growth. Shi et al. synthesized a novel scale and corrosion inhibitor—poly(aspartic acid) functionalized with furfurylamine [93]. The inhibition efficiency was close to 100% against CaCO_3_, about 96% against Ca_3_(PO_4_)_2_, and close to 30% for corrosion. Wu et al. developed a new non-phosphorous inhibitor against CaCO_3_ scale for cooling water systems glutamic-modified polyethercopolymer (AA-APEU) [94]. It was found that this inhibitor exhibited excellent ability to control CaCO_3_ scale with an efficiency of about 71.8%. Du et al. using one-step graft copolymerization prepared starch-*graft*-PAA polymer as a water treatment agent [95]. Its scale-inhibition efficiency against CaCO_3_ was equal approximately to 94%. The turbidity reduction was about 97% when it was used as a coagulant for poly-aluminum chloride (PAC) flocculation of hairwork wastewater. Nakao et al. investigated CaCO_3_ mineralization using cellulose nanocrystal/polymer composites [96]. Chen et al. synthesized a new inhibitor against CaCO_3_, which contains carboxylic acid and ether groups [97]. Its scale inhibition efficiency was 95.60% and showed good biodegradability performance. Chen et al. also prepared a modified polyepoxysuccinic acid (PESA) scale inhibitor and then applied it for the inhibition of such scales as CaCO_3_, CaSO_4_, and Ca_3_(PO_4_)_2_ in cooling water [98]. The polymer exhibited excellent scale inhibition properties against calcium scales. Zahlan et al. investigated the ability of hyperbranched polyesters involving citric acid and glycerol monomers to inhibit the deposition of inorganic scales [99]. The obtained polymer showed good inhibition efficiency at the elevated temperature, reaching 75% at 100 °C. Cui and Zhang prepared and tested a new non-phosphorus, multipurpose copolymer to control the formation of inorganic calcium scale and to improve the environmental benefits of coal water slurry [100]. The maximum scale inhibition efficiencies for CaCO_3_ and CaSO_4_ were about 94%. Wang et al. designed the conditioning process that combines crystallization of magnesium ammonium phosphate with organic polymer flocculation [101]. Cationic polyacrylamides formed flocs which were characterized by a larger size and a more compact structure. The proposed method reduces the ammonium nitrogen load in anaerobic digestion liquor and increases the suitability of biosolids as land fertilizers.

#### 3.3.2. Organic(-Inorganic) Electronics

Chen et al. showed that metastable polymorphs of 6,13-bis(triisopropylsilylethynyl)pentacene (TIPS-pentacene), obtained in the presence of conjugated polymer additives such as poly(3-hexylthiophene) (P3HT) and regiorandom pentacenebithiophene polymer (PnBT-RRa), can provide a novel design strategy for attaining high device performance in organic electronics [102]. Bi et al. prepared perovskite films of high electronic quality by using poly(methyl methacrylate) (PMMA) as a template to control nucleation and crystal growth [103]. Ren et al. reported the preparation of perovskite semiconductor CsPbBr_3_ film (used in photovoltaic devices) of improved quality via the introduction of PEG into solution [104]. Zhang et al. prepared cadmium sulfide nanodots using crystallization in a polyacrylamide colloidal reactor [105]. The obtained small cadmium sulfide crystals displayed the desired property of photocatalytic degradation. Ghosh et al. reported the crystallization of three forms of the copper (II) isonicotinate-*N*-oxide complex and their phase interconversion via solvent-mediated crystal-to-crystal transformation [106]. Lu et al. tried to explain the retardation mechanism on cement hydration using a highly carboxylated polystyrene latex [107]. Pan et al. studied the influence of the horizontal distribution of two polymers on the dispersion and crystallization of commonly used organic semiconductor—pentacene [108]. They found that the distribution of polymer phases is advantageous for the evolution of two-dimensional stacking of functional crystals. Purohit and Sistla tested a combination of porous polyurethane foam sheets and saturated inorganic salt solution as latent heat thermal storage systems [109]. Cividanes et al. reported the study of the effect of urea on mullite crystallization, synthesized through the colloidal and polymeric sol-gel processes [110]. The urea addition in colloidal gels led to materials with higher concentrations of orthorhombic mullite and, as a positive effect, mullite crystallized at lower temperatures. However, a negative effect was observed when urea was added to polymeric gels.

#### 3.3.3. Miscellaneous Applications

Recent years have shown that polymers are the most investigated materials for application in the biomedical field. Thus, it is good to mention publications in which polymers prevented or induced controlled crystallization of inorganic and low molecular weight organic compounds. Schweikle et al. performed the experiments in which amorphous Ca_3_(PO_4_)_2_ composite was formed in the synthetic PEG-based hydrogel matrix [111]. Such composites are promising biomaterials to replace human and animal-derived bone scaffolds. Adawy et al. investigated confined crystallization of inorganic compounds performed in bowl-shaped polymeric stomatocytes compartments which provided shielding from the electron beam destroying effects during TEM experiments [112]. Stubbs et al. used photochemical polymerization to obtain well-defined PVP copolymers to test the impact of hydrophobicity on ice recrystallization inhibition in a polymer system [113]. The obtained results can help to design new polymers for cryopreservation applications. Akshaykranth et al. in their experiments have grown zinc oxide nanorods on the bio-degradable PLA substrates using a low-temperature solution (70 °C) growth method. The obtained results can be useful in the preparation of environmentally friendly food packaging with antibacterial properties [114].

As was mentioned earlier, the influence of polymers on crystallization of other polymers is a very broad field; therefore, in this review just one exemplary publication was referred in order to give the reader the full picture of the topic. The same situation concerns the application of polymers in the crystallization of proteins. This phenomenon is very popular and intensively researched nowadays. Therefore, in order to give just the glimpse of the eye for the interested reader, a few publications will be mentioned here. Namely, Saridakis and Chayen in their review paper presented the role of molecularly imprinted polymers (MIPs) as nucleation-inducing substances for protein crystals [115]. Profio et al. demonstrated the fabrication of hydrogel membranes displaying controlled chemical composition and nanostructure in the creation of crystals with enhanced diffraction properties at a lower protein concentration than the conventional technique [116]. Belviso et al. used ionic-liquid hydrogel composite membranes as material for supporting protein crystallization [117]. They proved that protein crystallization by ionic-liquid hydrogel composite membranes had the potential for biotechnological applications and could contain crystallized enzymes in working conditions. Moreover, Bakshi wrote a review paper highlighting the relationship between polymeric surfactants’ properties and their ability in designing nanomorphology [118]. Such an approach can be implemented to prepare biologically sustainable nanomaterials for use in nanomedicine.

### 3.4. Discussion and Conclusions

**Figure 2:**
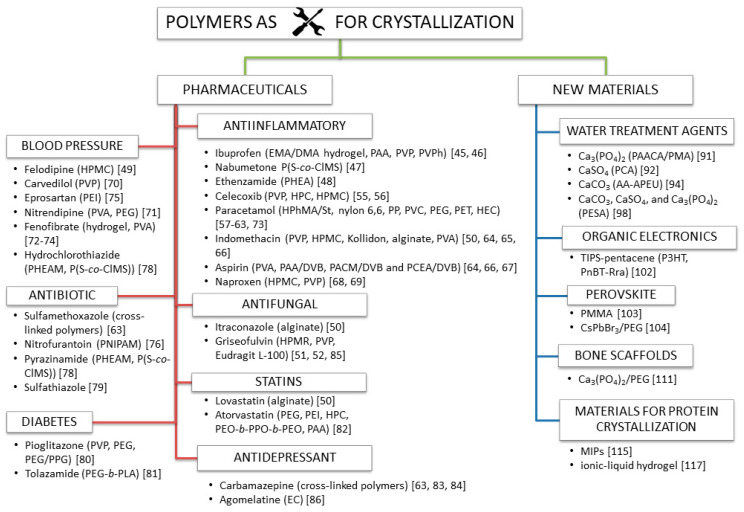
Utilization of polymers in drug amelioration and new material preparation.

Increasing research efforts have aimed to find economical and environmentally friendly inhibitors in industrial cooling water treatment. Scaling by CaCO_3_, CaSO_4_ and Ca_3_(PO_4_)_2_ precipitation is one of the major challenges in circulating cooling water systems. The abovementioned publications indicate that polymers can be used as efficient “green” scale inhibitors for calcium salts (Figure 2).

Recently, organic electronics appears to be a promising field, showing a wide range of applications, with such advantages as low production cost, versatility in material synthesis, and compatibility with flexible polymeric materials. Next, research into processes controlling the nucleation and growth of minerals is of major interest in the concrete and gypsum industries (Figure 2).

The past several years also witnessed the rapid development of solar cells based on mixed organic-inorganic halide perovskites. All-inorganic perovskite material (CsPbBr3) has attracted vast attention in photovoltaic devices due to its superior stability (Figure 2). 

Storage of energy from renewable sources such as wind, solar power, etc. is essential for daily energy needs. The application of phase change materials as latent heat thermal storage systems can solve these problems. 

Polymorphism in confined environments is poorly understood, even though nanoporous materials have been used for controlling the crystallization of polymorphs for years. Molecularly imprinted polymers polymerized in the presence of a template molecule of which they retain a chemical “memory” are regarded as “smart materials”. When the template molecule is extracted from the polymer, it leaves behind cavities, thus making the material capable of rebinding that molecule with high affinity and selectivity. The development of suitable materials for controlled heterogeneous nucleation can improve biomacromolecular crystallization in different conditions. Protein crystallization is a powerful purification tool, being the first step for crystallographic structural investigations, and can be also preparatory for biotechnological applications (Figure 2).

## 4. Summary and Conclusions

This short survey aimed to demonstrate the importance of a better understanding of the impact of polymer presence on the nucleation process and subsequent growth of crystals. Such influence can manifest in the course of crystallization and properties of resulting solid product. The possibility to retard or stop the crystallization process is of great importance in many cases. However, mutual interactions between polymers and crystallizing solutions are very individual in character and each case should be treated separately.

Firstly, papers discussing attempts to elucidate different aspects and mechanisms of polymer-induced and polymer-control crystallization are presented. Another chapter concerns papers reflecting the aim to improve the properties and behavior of the crystallizing drug via polymers’ application. Finally, works focused on the preparation of advanced materials such as polymer electrolytes, antiscalants, biologically sustainable nanomaterials, perovskite semiconductors and composite materials of different applications, are reported. In order to facilitate the reading of this text and to simplify the search for desired information, the tables comparing the polymer-crystallized substance systems are placed below. Table 1 is dedicated to confined crystallization, Table 2 presents the research concerning crystallization with use of polymeric surfaces, and Table 3 refers to crystallization within free polymer solutions.

In conclusion, the authors would like to emphasize that the branch of science tackling complicated interactions between crystallizing solutions and polymer additives becomes currently more and more important from both theoretical and practical points of view and therefore is a promising area for further research.

## Figures and Tables

**Table 1:** Confined crystallization: Polymers and crystallized substances.

Type of Process	Polymer Used as Tool	Crystallized Substance	Reference
Confined crystallization	Alginate	CaCO_3_	[12]
	Silk fibroin	CaCO_3_	[13]
	Gelatin	CaCO_3_	[14]
	Alginate, xanthan	CaCO_3_	[15]
	Polyacrylamide	Ca_3_(PO_4_)_2_	[16]
	Biopolymer	TiO_2_	[18]
	Poly(ethylene glycol) diacrylate	Aspirin, paracetamol	[19]
	Poly(ethylene glycol) diacrylate	Aspirin, paracetamol	[20]
	Glutaraldehyde crosslinked chitosan	Zeolite	[21]
	homopolymers and copolymers of nethylmorpholine methacrylamide and *N*,*N*-dimethylacrylamide	Ibuprofen	[45]
	Poly(ethylene glycol) diacrylate	Paracetamol	[57]
	Alginate	Fenofibrate	[72]
	Alginate	Paracetamol, fenofibrate	[73]
	Alginate	Fenofibrate	[74]
	2*H*-benzo[*d*]1,2,3-triazole derivatives	Theophylline, sulfathiazole, sulfamerazine and niflumic acid	[79]
	Tannic acid-Ti^IV^ metallogel	Caffeine, carbamazepine, piroxicam	[83]
	Poly(ethylene glycol) diacrylate	Carbamazepine, 5-methyl-2-[(2-nitrophenyl)amino]-3-thiophenecarbonitrile	[84]
	Agarose, tris-amide of trimesic acid with L-valine methyl ester, 3,3′,3″-((Benzene-1,3,5-tricarbonyl)tris(azanediyl))tris-(pyridine 1-oxide)	Copper(II) isonicotinate–*N*-oxide complex, Cu(NO_3_)_2_·3H_2_O	[106]
	Open cell polyurethane foam composites	Na_2_SO_4_, MgSO_4_·7H_2_O, and their mixture	[109]
	Polymer gels from urea, aluminum nitrate nonahydrate, tetraethylorthosilicate	Mullite	[110]
	Poly(ethylene glycol)	Ca_3_(PO_4_)_2_	[111]
	Stomatocytes	KH_2_PO_4_, NaCl, NaClO_3_, (NH_4_)_2_SO_4_	[112]

**Table 2:** Crystallization on polymeric surfaces/nucleation induction: Polymers and crystallized substances.

Type of Process	Polymer Used as Tool	Crystallized Substance	Reference
Crystallization on polymeric surfaces/ nucleation induction	Microporous poly(vinylidene fluoride) membranes	hen egg white lysozyme	[22]
	Poly(acrylic acid), poly(sodium 4-styrenesulfonate), poly(allylamine hydrochloride), poly(ethyleneimine), polyamide pellets	CaSO_4_·2H_2_O	[23]
	Polyethylene, polypropylene, polyvinyl chloride, poly(vinyl alcohol), polystyrene, poly(4-aminostyre)	Benzocaine, 1,1′-bi-2-naphthol	[24]
	Siloxane templates	Diphenylurea	[25]
	Polyethylene, polyvinyl chloride, polycarbonate, PET, nylon 66, ethylene vinyl alcohol, cellulose acetate, poly(methyl methacrylate)	CaCO_3_	[26]
	Nanocomposites	PET	[27]
	2-((4-vinylphenyl)amino)-benzoic acid/divinylbenzene copolymer, 2-((4-vinylphenyl)amino)-benzoic acid/divinylbenzene copolymer, hydroxyethyl methacrylate/divinylbenzene copolymer, hydroxyethyl methacrylate/divinylbenzene copolymer	paracetamol, mefenamic acid	[58]
	Nylon 66, polyester, polypropylene	paracetamol	[60]
	2-hydroxyethyl cellulose	paracetamol	[61]
	Functionalized Merrifield Resin	paracetamol	[62]
	Cross-linked terpolymers in which one component is the cross-linker	paracetamol, sulfamethoxazole, carbamazepine, 5-methyl2-[(2-nitrophenyl)amino]-3-thiophenecarbonitrile	[63]
	poly(vinyl alcohol)	Aspirin, indomethacin	[64]
	Poly(acrylic acid) films crosslinked with divinylbenzene	Aspirin	[66]
	Poly(4-acryloylmorpholine) and poly(2-carboxyethyl acrylate), each cross-linked with divinylbenzene	Aspirin	[67]
	poly(styrene-co-chloromethylstyrene)	Pyrazinamide, hydrochlorothiazide	[78]
	Ethyl cellulose	Agomelatine Form I	[86]
	cellulose nanocrystal/poly(2-hydroxyethyl methacrylate), cellulose nanocrystal/poly(2-hydroxyethyl methacrylate-co-acrylic acid)	CaCO_3_	[96]
	Poly(methyl methacrylate)	Pervoskite	[103]
	Polyacrylamide	CdS	[105]
	Highly carboxylated polystyrene latex	Calcium silicate hydrate	[107]
	Poly(lactic acid)	ZnO	[114]
	Acrylamide, poly(ethylene glycol)dimethacrylate	Lysozyme, concanavalin A	[116]

**Table 3:** Crystal surface screening/nucleation inhibition: Polymers and crystallized substances.

Crystal surface screening/nucleation inhibition	Poly(sodium 4-styrenesulfonate)	ZnO	[28]
	Poly(vinyl alcohol), polyvinylpyrolidone	ZnO	[29]
	Molecularly imprinted polymers (oleanolic acid as template, acrylamide as functional monomer, ethylene glycol dimethacrylate as cross-linker and azobisisobutyronitrile as initiator)	Oleanolic acid	[30]
	Polyacrylamide, poly(vinyl alcohol), poly(ethylene glycol), poly-*N*-vinyl pyrrolidone	PbS	[31]
	Maleic acid copolymers	Calcium orthophosphates	[32]
	Poly(ethylene oxide)	Copper/poly(ethylene oxide)/low density polyethylene composite	[33]
	Guar gum, pectin, κ-carrageenan, gelatin, polyvinylpyrrolidone	Thiamine chloride hydrochloride	[34]
	Poly(acrylic acid), poly(*N*-vinyl pyrrolidone), poly(4-vinylphenol)	Ibuprofen	[46]
	Polymers functionalized from a parent batch of poly(chloromethylstyrene-co-styrene)	Nabumetone	[47]
	Functionalized poly(*N*-hydroxyethyl acrylamide)	Ethenzamide	[48]
	Hydroxypropylmethyl cellulose	Felodipine	[49]
	Alginate	Lovastatin, indomethacin, itraconazole	[50]
	Hydroxypropyl methylcellulose and methylcellulose polymers	Griseofulvin	[51]
	Hydroxypropyl methylcellulose, polyvinylpyrrolidone), Eudragit L-100	Griseofulvin, danazol	[52]
	Different polymers of vinylpyrrolidone and a copolymer of vinylpyrrolidone and vinylacetate	Sixteen drugs of different chemical nature	[53]
	Polyvinylpyrrolidone	Celecoxib	[55,56]
	Nylon 6/6, polypropylene, polyvinylchloride	paracetamol	[59]
	Polyvinylpyrrolidone, hydroxypropyl methyl cellulose, Kollidone VA64	Indomethacin	[65]
	Hydroxypropyl methylcellulose, polyvinylpyrrolidone	Naproxen	[68]
	Polyvinylpyrrolidone	Naproxen	[69]
	Polyvinylpyrrolidone	Carvedilol	[70]
	Poly(vinyl alcohol), polyethylene glycol	Nitrendipine	[71]
	Poly(ethylene imine)	Eprosartan	[75]
	Poly(*N*-isopropyl acrylamide)	Nitrofurantoin	[76]
	Hydroxypropylmethyl cellulose, copovidone	Complex impurity	[77]
	Polyvinylpolypyrrolidone K30 and K90, polyethyleneglycol 6000, polyethylene-polypropylene glycol 188	Pioglitazone	[80]
	Poly(ethylene glycol)-block-poly(lactic acid)	Tolazamide	[81]
	Poly(ethylene glycol), poly(ethylene oxide)-poly(propylene oxide) triblock, hydroxypropyl cellulose, poly(acrylic acid), poly(ethylene imine), elastin-like peptide, chitosan	Atorvastatin calcium	[82]
	Eudragit RL100	Griseofulvin	[85]
	Poly(aspartic acid-citric acid) copolymer, polymaleic acid	Ca_3_(PO4)_2_	[91]
	Poly(citric acid)	CaSO_4_	[92]
	Polyaspartic acid/furfurylamine graft copolymer	CaCO_3_, Ca_3_(PO4)_2_	[93]
	Acrylic acid-allyloxy polyethoxy glutamate copolymer	CaCO_3_	[94]
	Starch-graft-poly(acrylic acid)	CaCO_3_	[95]
	Epoxysuccinic acid derivative	CaCO_3_	[97]
	Epoxysuccinic acid derivative	CaCO_3_, CaSO_4_, Ca_3_(PO_4_)_2_	[98]
	Poly(Citric Acid-*co*-Glycerol)	CaCO_3_	[99]
	Itaconic acid-2-acrylamido-2-methylpropanesulfonic acid allyl polyoxyethylene ether copolymer	CaCO_3_	[100]
	Organic polymer	Struvit	[101]
	Regiorandom pentacene-bithiophene, poly(3-hexylthiophene)	6,13-bis(triisopropylsilylethynyl)-pentacene	[102]
	Poly(ethylene glycol)	Perovskite	[104]
	Poly(3-hexylthiophene), poly(methyl methacrylate)	Bis(triisopropylsilylethynyl) pentacene	[108]
	Maleic acid and sodium ρ-styrenesulfonate copolymer	CaSO_4_·2H_2_O	[109]
	Poly(*N*-vinyl pyrrolidone) copolymers	H_2_O	[113]
	Composite membranes	Glucose Isomerase	[117]

## References

[1] Bałdyga J., Tyl G., Bouaifi M. (2017). Application of Gaussian cubature to model two-dimensional population balances. Chem. Process Eng..

[2] Silva J.E., Paiva A.P., Soares D., Labrincha A., Castro F., Edward C. (2007). Plating Sludge Value-Adding by Application of Hydrometallurgical Processes. Trends in Hazardous Materials Research.

[3] Geddert T., Augustin W., Scholl S. (2011). Induction Time in Crystallization Fouling on Heat Transfer Surfaces. Chem. Eng. Technol..

[4] Herz A., Malayeri M.R., Müller-Steinhagen H. (2008). Fouling of roughened stainless steel surfaces during convective heat transfer to aqueous solutions. Energy Convers. Manag..

[5] Bogacz W., Lemanowicz M., Al-Rashed M.H., Nakonieczny D., Piotrowski T., Wójcik J. (2017). Impact of roughness, wettability and hydrodynamic conditions on the incrustation on stainless steel surfaces. Appl. Therm. Eng..

[6] Gao Z., Rohani S., Gong J., Wang J. (2017). Recent Developments in the Crystallization Process: Toward the Pharmaceutical Industry. Engineering.

[7] Bauer J.F., Saleki-Gerhardt A., Narayanan B.A., Chemburkar S.R., Patel K.M., Spiwek H.O., Bauer P.E., Allen K.A. (2014). Polymorph of a Pharmaceutical. US Patent.

[8] Bauer J., Spanton S., Henry R., Quick J., Dziki W., Porter W., Morris J. (2001). Ritonavir: An Etraordinary Example of Conformational Polymorphism. Pharm. Res..

[9] Mullin J.W. (2001). Crystallization.

[10] Chernov A.A. (1989). Formation of crystals in solutions. Contemp. Phys..

[11] Lovette M.A., Browning A.R., Griffin D.W., Sizemore J.P., Snyder R.C., Doherty M.F. (2008). Crystal Shape Engineering. Ind. Eng. Chem. Res..

[12] Ma Y., Feng Q. (2011). Alginate hydrogel-mediated crystallization of calcium carbonate. J. Solid State Chem..

[13] Ma Y., Feng Q., Bourrat X. (2013). A novel growth process of calcium carbonate crystals in silk fibroin hydrogel system. Mater. Sci. Eng. C.

[14] Nindiyasari F., Fernandez-Diaz L., Griesshaber E., Manuel A.J., Sanchez-Pastor N., Schmahl W.W. (2014). Influence of Gelatin Hydrogel Porosity on the Crystallization of CaCO_3_. Cryst. Growth Des..

[15] Kosanovic C., Fermani S., Falini G., Kralj D. (2017). Crystallization of Calcium Carbonate in Alginate and Xanthan Hydrogels. Crystals.

[16] Yokoi T., Kawashita M., Kikuta K., Ohtsuki C. (2010). Crystallization of calcium phosphate in polyacrylamide hydrogels containing phosphate ions. J. Cryst. Growth.

[17] Velasquez-Gonzalez O., Campos-Escamilla C., Flores-Ibarra A., Esturau-Escofet N., Arreguin-Espinosa R., Stojanoff V., Cuellar-Cruz M., Moreno A. (2019). Crystal Growth in Gels froms the Mechanisms of Crystal Growth to Controlof Polymorphism: New Trends on Theoretical and Experimental Aspects. Crystals.

[18] Choi D., Sonkaria S., Fox S.J., Poudel S., Kim S.Y., Kang S., Kim S., Verma C., Ahn S.H., Lee C.S. (2019). Quantum scale biomimicry of low dimensional growth: An unusual complex amorphous precursor route to TiO2 band confinement by shape adaptive biopolymer-like flexibility for energy applications. Sci. Rep..

[19] Diao Y., Helgeson M.E., Myerson A.S., Hatton T.A., Doyle P.S., Trout B.L. (2011). Controlled Nucleation from Solution Using Polymer Microgels. J. Am. Chem. Soc..

[20] Diao Y., Helgeson M.E., Siam Z.A., Doyle P.S., Myerson A.S., Hatton T.A., Trout B.L. (2012). Nucleation under Soft Confinement: Role of Polymer-Solute Interactions. Cryst. Growth Des..

[21] Li D., Huang Y., Ratinac K.R., Ringer S.P., Wang H. (2008). Zeolite crystallization in crosslinked chitosan hydrogels: Crystal size control and chitosan removal. Microporous Mesoporous Mater..

[22] Curcio E., Fontananova E., Di Profio G., Drioli E. (2006). Influence of the Structural Properties of Poly(vinylidene fluoride) Membranes on the Heterogeneous Nucleation Rate of Protein Crystals. J. Phys. Chem. B.

[23] Lin N.H., Shih W.-Y., Lyster E., Cohen Y. (2011). Crystallization of calcium sulfate on polymeric surfaces. J. Colloid Interface Sci..

[24] Patel M.A., Nguyen B., Chadwick K. (2017). Predicting the Nucleation Induction Time Based on Preferred Intermolecular Interactions. Cryst. Growth Des..

[25] Solomos M.A., Capacci-Daniel C., Rubinson J.F., Swift J.A. (2018). Polymorph Selection via Sublimation onto Siloxane Templates. Cryst. Growth Des..

[26] Hutfles J., Ravichandran S.A., Pellegrino J. (2019). Screening polymer surfaces in crystallization. Colloids Surf. A Physicochem. Eng. Asp..

[27] Xing S., Li R., Si J., Tang P. (2016). In situ polymerization of poly(styrene-alt-maleic anhydride)/organic montmorillonite nanocomposites and their ionomers as crystallization nucleating agents for poly(ethylene terephthalate). J. Ind. Eng. Chem..

[28] Yue S., Zhang L., Lu J., Zhang J. (2009). Polymer-controlled crystallization of dumbbell-like ZnO hollow architectures. Mater. Lett..

[29] Miculescu F., Rusen E., Mocanu A., Diacon A., Birjega R. (2013). Hierarchical nanostructures of ZnO obtained in the presence of water soluble polymers. Powder Technol..

[30] Zhang W., Zhang H., Zhang Q., Cui Y., Wu Z., Zheng R., Liu L. (2011). Molecularly imprinted polymers prepared by precipitation polymerizationand used for inducing crystallization of oleanolic acid in supercritical CO_2_. Sep. Purif. Technol..

[31] Preda N., Rusen E., Enculescu M., Matei E., Marculescu B., Enculescu I. (2011). Polymer-assisted crystallization of low-dimensional lead sulfide particles. Phys. E Low Dimens. Syst. Nanostruct..

[32] Pelin I.M., Popescu I., Suflet D.M., Aflori M., Bulacovschi V. (2013). Influence of maleic acid copolymers on calcium orthophosphates crystallization at low temperature. J. Cryst. Growth.

[33] Lu Y., Tang Y., Xia X. (2018). Non-isothermal crystallization of copper-containing composite based on polymer alloy of poly(ethylene oxide) and polyethylene. Thermochim. Acta.

[34] Arioglu-Tuncil S., Bhardwaj V., Taylor L.S., Mauer L.J. (2017). Amorphization of thiamine chloride hydrochloride: A study of the crystallization inhibitor properties of different polymers in thiamine chloride hydrochloride amorphous solid dispersions. Food Res. Int..

[35] Sinek A., Kupczak M., Mielańczyk A., Lemanowicz M., Yusa S., Neugebauer D., Gierczycki A. (2020). Temperature and pH-Dependent Response of Poly(Acrylic Acid) and Poly(Acrylic Acid-co-Methyl Acrylate) in Highly Concentrated Potassium Chloride Aqueous Solutions. Polymers.

[36] Beyer R., Iacopini S., Palberg T., Schoepe H.J. (2012). Polymer induced changes of the crystallization scenario in suspensionsof hard sphere like microgel particles. J. Chem. Phys..

[37] Marinović-Cincović M., Janković B., Milićević B., Antić Ž., Whiffen R.K., Dramićanin M.D. (2013). The comparative kinetic analysis of the non-isothermal crystallization process of Eu3 + doped Zn2SiO4 powders prepared via polymer induced sol–gel method. Powder Technol..

[38] Sun H., Luo Y., Yang B., Zhang H., Huang J. (2018). Non-isothermal crystallization of biopolyesters of poly(butylenesuccinate) formed via in-situ polymerization in presence of poly(vinylbutyral). Polymer.

[39] Liu K., Yuan Y., Zhang J. (2011). Isothermal crystallization behavior of water in poly(vinyl methyl ether) aqueous solution investigated by infrared and two-dimensional infrared correlation spectroscopy. Vib. Spectrosc..

[40] Mandal T., Huang W., Mecca J.M., Getchell A., Porter W.W., Larson R.G. (2017). A framework for multi-scale simulation of crystal growth in the presenceof polymers. Soft Matter.

[41] Yan Y., Zhang R., Liang Q., Liu J., Han Y. (2019). Control the interplay of crystallization and phase separation of conjugated polymer blends by the relative rate of nucleation and growth. Polymer.

[42] Olmstead M.M., Ghiassi K.B., Constable E.C., Parkin G., Que L. (2021). Crystal Growth and Crystal Transformation. Comprehensive Coordination Chemistry.

[43] Bakshi M.S., Thakur P., Sachar S., Kaur G., Banipal T.S., Possmayer F., Petersen N.O. (2007). Aqueous phase surfactant selective shape controlled synthesis of lead sulfide nanocrystals. J. Phys. Chem. C.

[44] The Cambridge Crystallographic Data Centre the Cambridge Structural Database (CSD). https://www.ccdc.cam.ac.uk/solutions/csd-core/components/csd/.

[45] Velasco D., Danoux C.B., Redondo J.A., Elvira C., SanRoman J., Wray P.S., Kazarian S.G. (2011). pH-sensitive polymer hydrogels derived from morpholine to prevent thecrystallization of ibuprofen. J. Control. RELEASE.

[46] Lee S.Y., Yu G., Kim I.W. (2013). Effects of polymeric additives on the crystallization and release behavior of amorphous ibuprofen. J. Nanomater..

[47] Frank D.S., Matzger A.J. (2018). Probing the Interplay between Amorphous Solid Dispersion Stability and Polymer Functionality. Mol. Pharm..

[48] Frank D.S., Matzger A.J. (2019). Effect of Polymer Hydrophobicity on the Stability of Amorphous Solid Dispersions and Supersaturated Solutions of a Hydrophobic Pharmaceutical. Mol. Pharm..

[49] Alonzo D.E., Raina S., Zhou D., Gao Y., Zhang G.G.Z., Taylor L.S. (2012). Characterizing the impact of hydroxypropylmethyl cellulose on the growth and nucleation kinetics of felodipine from supersaturated solutions. Cryst. Growth Des..

[50] Guan J., Liu Q., Liu J., Cui Z., Zhang X., Mao S. (2020). Elucidation of alginate-drug miscibility on its crystal growth inhibition effect in supersaturated drug delivery system. Carbohydr. Polym..

[51] Hong S., Nowak S.A., Wah C.L. (2018). Impact of Physicochemical Properties of Cellulosic Polymers on Supersaturation Maintenance in Aqueous Drug Solutions. AAPS PharmSciTech.

[52] Ozaki S., Kushida I., Yamashita T., Hasebe T., Shirai O., Kano K. (2013). Inhibition of crystal nucleation and growth by water-soluble polymers and its impact on the supersaturation profiles of amorphous drugs. J. Pharm. Sci..

[53] Fornells E., Fuguet E., Mañé M., Ruiz R., Box K., Bosch E., Ràfols C. (2018). Effect of vinylpyrrolidone polymers on the solubility and supersaturation of drugs; a study using the Cheqsol method. Eur. J. Pharm. Sci..

[54] Chavan R.B., Rathi S., Jyothi V.G.S.S., Shastri N.R. (2019). Cellulose based polymers in development of amorphous solid dispersions. Asian J. Pharm. Sci..

[55] Lee H., Lee J. (2013). Dissolution enhancement of celecoxib via polymer-induced crystallization. J. Cryst. Growth.

[56] Sodhi I., Mallepogu P., Thorat V.P., Kashyap M.C., Sangamwar A.T. (2019). Insights on role of polymers in precipitation of celecoxib from supersaturated solutions as assessed by focused beam reflectance measurement (FBRM). Eur. J. Pharm. Sci..

[57] Sharma M., Trout B.L. (2015). Effect of Pore Size and Interactions on Paracetamol Aggregation in Porous Polyethylene Glycol Diacrylate Polymers. J. Phys. Chem. B.

[58] Pfund L., Price C., Frick J., Matzger A. (2014). Controlling Pharmaceutical Crystallization with Designed Polymeric Heteronuclei. J. Am. Chem. Soc..

[59] Sudha C., Nandhini R., Srinivasan K. (2014). Polymer-Induced Selective Nucleation of Mono or Ortho Polymorphs of Paracetamol through Swift Cooling of Boiled Aqueous Solution. Cryst. Growth Des..

[60] Song Y., Cai Z., Li Z., Guan G., Jiang Y. (2018). Preferential Orientation Effect of Polymers on Paracetamol Crystallization: Experiments and Modeling. Cryst. Growth Des..

[61] Stojaković J., Baftizadeh F., Bellucci M.A., Myerson A.S., Trout B.L. (2017). Angle-Directed Nucleation of Paracetamol on Biocompatible Nanoimprinted Polymers. Cryst. Growth Des..

[62] Frank D.S., Matzger A.J. (2017). Influence of Chemical Functionality on the Rate of Polymer-InducedHeteronucleation. Cryst. Growth Des..

[63] Price C.P., Grzesiak A.L., Matzger A.J. (2005). Crystalline Polymorph Selection and Discovery with Polymer Heteronuclei. J. Am. Chem. Soc..

[64] Tan L., Davis R.M., Myerson A.S., Trout B.L. (2015). Control of Heterogeneous Nucleation via Rationally Designed Biocompatible Polymer Surfaces with Nanoscale Features. Cryst. Growth Des..

[65] Cheng H., Mao L., Zhang S., Lv H. (2019). Impacts of Polymeric Additives on Nucleation and Crystal Growth of Indomethacin from Supersaturated Solutions. AAPS PharmSciTech.

[66] Diao Y., Harada T., Myerson A., Hatton T., Trout B. (2011). The role of nanopore shape in surface-induced crystallization. Nat. Mater..

[67] Diao Y., Myerson A.S., Hatton T.A., Trout B.L. (2011). Surface Design for Controlled Crystallization: The Role of Surface Chemistry and Nanoscale Pores in Heterogeneous Nucleation. Langmuir.

[68] Poornachary S.K., Chia V.D., Yani Y., Han G., Chow P.S., Tan R.B.H. (2017). Anisotropic Crystal Growth Inhibition by Polymeric Additives: Impact on Modulation of Naproxen Crystal Shape and Size. Cryst. Growth Des..

[69] Gupta K.M., Yani Y., Poornachary S.K., Chow P.S. (2019). Atomistic Simulation To Understand Anisotropic Growth Behavior ofNaproxen Crystal in the Presence of Polymeric Additives. Cryst. Growth Des..

[70] Pataki H., Sóti P., Vigh T., Nagy Z., Vajna B., Csontos I., Marosi G. (2014). Controlled Formation of Free-Flowing Carvedilol Particles in the Presence of Polyvinylpyrrolidone. Chem. Eng. Technol..

[71] Xia D., Wu J.X., Cui F., Qu H., Rades T., Rantanen J., Yang M. (2012). Solvent-mediated amorphous-to-crystalline transformation of nitrendipine in amorphous particle suspensions containing polymers. Eur. J. Pharm. Sci..

[72] Eral H.B., O’Mahony M., Shaw R., Trout B.L., Myerson A.S., Doyle P.S. (2014). Composite Hydrogels Laden with Crystalline Active Pharmaceutical Ingredients of Controlled Size and Loading. Chem. Mater..

[73] Eral H.B., López-Mejías V., O’Mahony M., Trout B.L., Myerson A.S., Doyle P.S. (2014). Biocompatible Alginate Microgel Particles as Heteronucleants and Encapsulating Vehicles for Hydrophilic and Hydrophobic Drugs. Cryst. Growth Des..

[74] Badruddoza A.Z., Godfrin P., Myerson A., Trout B., Doyle P. (2016). Core–Shell Composite Hydrogels for Controlled Nanocrystal Formation and Release of Hydrophobic Active Pharmaceutical Ingredients. Adv. Healthc. Mater..

[75] Bae H., Lee J. (2015). Morphology control of eprosartan crystals via polymer-directed crystallization. J. Pharm. Investig..

[76] Munk T., Baldursdottir S., Hietala S., Rades T., Kapp S., Nuopponen M., Kalliomäki K., Tenhu H., Rantanen J. (2012). Crystal Morphology Modification by the Addition of Tailor-Made Stereocontrolled Poly(*N*-isopropyl acrylamide). Mol. Pharm..

[77] Czyzewski A.M., Chen S., Bhamidi V., Yu S., Marsden I., Ding C., Becker C., Napier J.J. (2017). Use of a Polymer Additive to Enhance Impurity Rejection in the Crystallization of a Pharmaceutical Compound. Org. Process Res. Dev..

[78] Frank D.S., Zhu Q., Matzger A.J. (2019). Inhibiting or Accelerating Crystallization of Pharmaceuticals by Manipulating Polymer Solubility. Mol. Pharm..

[79] Torres-Moya I., Saikia B., Prieto P., Carrillo J.R., Steed J.W. (2019). High thermal stability, pH responsive organogels of 2*H*-benzo[*d*]1,2,3-triazole derivatives as pharmaceutical crystallization media. CrystEngComm.

[80] Shi N.-Q., Lei Y.-S., Song L.-M., Yao J., Zhang X.-B., Wang X.-L. (2013). Impact of amorphous and semicrystalline polymers on the dissolution andcrystallization inhibition of pioglitazone solid dispersions. POWDER Technol..

[81] Gao Y., Olsen K.W. (2015). Drug-Polymer Interactions at Water-Crystal Interfaces and Implicationsfor Crystallization Inhibition: Molecular Dynamics Simulations ofAmphiphilic Block Copolymer Interactions with Tolazamide Crystals. J. Pharm. Sci..

[82] Choi H., Lee H., Lee M.K., Lee J. (2012). Polymer-Directed Crystallization of Atorvastatin. J. Pharm. Sci..

[83] Rahim M.A., Hata Y., Bjornmalm M., Ju Y., Caruso F. (2018). Supramolecular Metal-Phenolic Gels for the Crystallization of ActivePharmaceutical Ingredients. SMALL.

[84] Diao Y., Whaley K.E., Helgeson M.E., Woldeyes M.A., Doyle P.S., Myerson A.S., Hatton T.A., Trout B.L. (2012). Gel-Induced Selective Crystallization of Polymorphs. J. Am. Chem. Soc..

[85] Chen D., Singh D., Sirkar K.K., Pfeffer R. (2016). Continuous preparation of polymer coated drug crystals by solid hollow fiber membrane-based cooling crystallization. Int. J. Pharm..

[86] Shi X., Hu S., Song S., Ding Z., Sheng X. (2019). Selective crystallization of agomelatine from molten state induced bypolymer. J. Drug Deliv. Sci. Technol..

[87] Parambil J.V., Poornachary S.K., Heng J.Y.Y., Tan R.B.H. (2019). Template-induced nucleation for controlling crystal polymorphism: From molecular mechanisms to applications in pharmaceutical processing. CrystEngComm.

[88] Thakore S.D., Sood A., Bansal A.K. (2020). Emerging role of primary heterogeneous nucleation in pharmaceutical crystallization. Drug Dev. Res..

[89] Sood J., Sapra B., Bhandari S., Tiwary A. (2015). Understanding pharmaceutical polymorphic transformations II: Crystallization variables and influence on dosage forms. Ther. Deliv..

[90] Wang C., Shen T., Li S., Wang X. (2014). Investigation of influence of low phosphorous co-polymer antiscalant on calcium sulfate dihydrate crystal morphologies. Desalination.

[91] Zhang Y.L., Zhao C.X., Liu X.D., Li W., Wang J.L., Hu Z.G. (2016). Application of poly(aspartic acid-citric acid) copolymer compound inhibitor as an effective and environmental agent against calcium phosphate in cooling water systems. J. Appl. Res. Technol..

[92] Zhao Y., Jia L., Liu K., Gao P., Ge H., Fu L. (2016). Inhibition of calcium sulfate scale by poly (citric acid). Desalination.

[93] Shi S., Zhao X., Wang Q., Shan H., Xu Y. (2016). Synthesis and evaluation of polyaspartic acid/furfurylamine graft copolymer as scale and corrosion inhibitor. RSC Adv..

[94] Wu Z., Zhou Y., Yao Q., Wang H., Liu Y., Tao W., Chu X., Sun W., Wu W. (2016). Synthesis of glutamic-modified polyether copolymer as a novel non-phosphorous inhibitor for calcium carbonate scales in cooling water systems. Desalin. Water Treat..

[95] Du Q., Wang Y., Li A., Yang H. (2018). Scale-inhibition and flocculation dual-functionality of poly(acrylic acid) grafted starch. J. Environ. Manag..

[96] Nakao Y., Sugimura K., Nishio Y. (2019). CaCO_3_ mineralization in polymer composites with cellulose nanocrystals providing a chiral nematic mesomorphic structure. Int. J. Biol. Macromol..

[97] Chen Y., Zhou Y., Yao Q., Nan Q., Zhang M., Sun W. (2019). Inhibition and biodegradability performance of modified polyepoxysuccinic acid as a scale inhibitor against calcium carbonate. Desalin. Water Treat..

[98] Chen Y., Zhou Y., Yao Q., Nan Q., Zhang M., Sun W. (2019). Synthesis of modified polyepoxysuccinic acid and evaluation of its scale inhibition on CaCO3, CaSO4, and Ca3(PO4)2 precipitation for industrial recycling water. Desalin. Water Treat..

[99] Zahlan H., Saeed W.S., Alrasheed R., Alandes N.M., Aouak T. (2019). Synthesis of poly (citric acid-co-glycerol) and its application as an inhibitor of CaCO3 deposition. Materials.

[100] Cui C., Zhang S. (2019). Synthesis, scale inhibition and dispersion performance evaluation of the environmentally benign additive IA-AMPS-APEG copolymer. Environ. Sci. Water Res. Technol..

[101] Wang Q., Zhang W., Yang Z., Xu Q., Yang P., Wang D. (2018). Enhancement of anaerobic digestion sludge dewatering performance using in-situ crystallization in combination with cationic organic polymers flocculation. Water Res..

[102] Chen J., Shao M., Xiao K., He Z., Li D., Lokitz B.S., Hensley D.K., Kilbey S.M., Anthony J.E., Keum J.K. (2013). Conjugated Polymer-Mediated Polymorphism of a High Performance, Small-Molecule Organic Semiconductor with Tuned Intermolecular Interactions, Enhanced Long-Range Order, and Charge Transport. Chem. Mater..

[103] Bi D., Yi C., Luo J., Decoppet Jean-Davidand Zhang F., Zakeeruddin S.M., Li X., Hagfeldt A., Gratzel M. (2016). Polymer-templated nucleation and crystal growth of perovskite films forsolar cells with efficiency greater than 21%. Nat. Energy.

[104] Ren Y., Hao Y., Zhang N., Arain Z., Mateen M., Sun Y., Shi P., Cai M., Dai S. (2019). Exploration of Polymer-Assisted Crystallization Kinetics in CsPbBr 3 All-Inorganic solar cell. Chem. Eng. J..

[105] Zhang Z., Xie B., Li J., Fang B., Lin Y. (2018). CdS nanodots preparation and crystallization in a polymeric colloidal nanoreactor and their characterizations. Colloids Surf. A Physicochem. Eng. Asp..

[106] Ghosh D., Ferfolja K., Drabavicius Z., Steed J.W., Damodaran K.K. (2018). Crystal habit modification of Cu(II) isonicotinate-*N*-oxide complexesusing gel phase crystallisation. NEW J. Chem..

[107] Lu Z., Kong X., Zhang C., Cai Y. (2018). Effect of highly carboxylated colloidal polymers on cement hydration and interactions with calcium ions. Cem. Concr. Res..

[108] Pan J.-H., Wu C.-F., Wang C.-A., Lin K.-T., Ruan J. (2018). Influence of horizontal distribution of polymer phases on the dispersion and crystallization of organic semiconductor triisopropylsilyl pentacene. Mater. Chem. Phys..

[109] Purohit B.K., Sistla V.S. (2017). Crystallization of inorganic salt hydrates in polymeric foam for thermalenergy storage application. J. Energy Storage.

[110] Cividanes L.S., Campos T.M.B., Bertran C.A., Brunelli D.D., Thim G.P. (2010). Effect of urea on the mullite crystallization. J. Non. Cryst. Solids.

[111] Schweikle M., Bjørnøy S.H., van Helvoort A.T.J., Haugen H.J., Sikorski P., Tiainen H. (2019). Stabilisation of amorphous calcium phosphate in polyethylene glycol hydrogels. Acta Biomater..

[112] Adawy A., Amghouz Z., van Hest J.C.M., Wilson D.A. (2017). Sub-Micron Polymeric Stomatocytes as Promising Templates for ConfinedCrystallization and Diffraction Experiments. SMALL.

[113] Stubbs C., Congdon T.R., Gibson M.I. (2019). Photo-polymerisation and study of the ice recrystallisation inhibition of hydrophobically modified poly(vinyl pyrrolidone) co-polymers. Eur. Polym. J..

[114] Akshaykranth A., Rao T.V., Kumar R.R. (2020). Growth of ZnO nanorods on biodegradable poly (lactic acid) (PLA) substrates by low temperature solution method. Mater. Lett..

[115] Saridakis E., Chayen N.E. (2013). Imprinted polymers assisting protein crystallization. Trends Biotechnol..

[116] Di Profio G., Polino M., Nicoletta F.P., Belviso B.D., Caliandro R., Fontananova E., De Filpo G., Curcio E., Drioli E. (2014). Tailored Hydrogel Membranes for Efficient Protein Crystallization. Adv. Funct. Mater..

[117] Belviso B.D., Caliandro R., Salehi Shabnam Majidiand Di Profio G., Caliandro R. (2019). Protein Crystallization in Ionic-Liquid Hydrogel Composite Membranes. Crystals.

[118] Bakshi M.S. (2016). How Surfactants Control Crystal Growth of Nanomaterials. Cryst. Growth Des..

